# Complete Coding Genome Sequence of an Influenza A/H3N8 Equine Virus Isolated in Kazakhstan in 2007

**DOI:** 10.1128/mra.01147-21

**Published:** 2022-09-12

**Authors:** Yerbol Burashev, Mukhit Orynbayev, Kunsulu Zakarya, Yergali Abduraimov, Markhabat Kassenov, Vitaliy Strochkov, Nurlan Kozhabergenov, Bekbolat Usserbayev, Aibarys Melisbek, Meirzhan Shirinbekov, Nuradyl Sypatay, Kulyaisan Sultankulova

**Affiliations:** a Research Institute for Biological Safety Problems (RIBSP), Gvardeyskiy, Kazakhstan; b Kazakh National Agrarian University, Almaty, Kazakhstan; c Faculty of Biology and Biotechnology, Al-Farabi Kazakh National University, Almaty, Kazakhstan; Portland State University

## Abstract

Here, we reported the complete coding sequence of the influenza A/equine/Otar/3/2007 (H3N8) equine virus, first isolated in Kazakhstan in 2007. The hemagglutinin (HA) sequences of the Kazakhstan isolates appeared to be closely related to viruses isolated in early 2000 in Asia. Phylogenetic analysis characterized the Kazakhstan isolates as a member of the Florida sublineage clade 2 by the HA protein sequence.

## ANNOUNCEMENT

Equine influenza (EI) is an infectious, acutely contagious disease of the equine family (horses, donkeys, mules, and zebras) (1, 2). The causative agent of EI is an RNA-containing virus belonging to the *Orthomyxoviridae* family and is 80 to 120 nm in diameter (3, 4). Due to the segmentation of the genome, influenza viruses often undergo rearrangement, which leads to reassortment and antigenic variability. The transfer of the virus or its genes into the animal population contributes to the preservation of the causative agent of influenza (5, 6).

It is assumed that the emergence of new pandemic strains occurs because of the reassortment of genes of human and animal influenza viruses. This process is easily reproduced in the laboratory and observed in nature. After every major human influenza epidemic, the corresponding viruses are found in animal populations.

The outbreaks of equine influenza in 2007 and 2012 in Kazakhstan were detected almost at the same time in China and Mongolia (7, 8).

Viral RNAs from nasal swabs were extracted using the QIAmp viral RNA extraction kit (Qiagen) according to the manufacturer’s instructions. Sequencing and amplification of eight segments of the virus genome were amplified in the SuperScript one-step reverse transcriptase PCR (RT-PCR) system with Platinum *Taq* DNA polymerase (Invitrogen SRL) using the Uni-12 (3-UCGYUUUCGUCC) and Uni-13 (GGAACAAAGAUGA-5) universal influenza primers (9). PCR products were cloned into pJEM plasmids and sequenced using m13 primers. Genome sequencing PCR products were analyzed on a 1.5% agarose gel stained with ethidium bromide and purified using the QIAquick PCR purification kit (Qiagen) according to the manufacturer’s instructions.

Sequencing was performed with a 16-capillary genetic analyzer AB3130xl automatic sequencher (Hitachi Applied Biosystems) using the BigDye Terminator version 3.1 cycle sequencing kit (ABI, Foster City, CA, USA). Chromatograms were edited and assembled using Sequencer version 5 (Gene Codes Corp.). Alignment of the nucleotide sequence with quality scores above 90% was carried out using the BioEdit version 7.2.5 program (https://bioedit.software.informer.com/7.2/).

The data were searched in the GenBank nucleotide (nt) database. The size of each virus segment and identity with the closest strains is shown in Table 1 according to the BLAST software (10).

**TABLE 1:** Genome characteristics of strain influenza A/equine/Otar/3/2007

Gene/ segment	Size (nucleotides)	GC content (%)	Strain with closest relative sequence	Identity at the nucleotide level (%)	GenBank accession no. (NCBI nt)
PB2	2341	42.5	A/equine/Himachal Pradesh/CMVL-YOL2/2008	99.7	MT908915
PB1	2341	42.1	A/equine/Richmond/1/2007	99.9	MT908914
PA	2084	41	A/equine/Richmond/1/2007	99.2	MT965694
HA	1733	39.4	A/equine/Richmond/1/2007	99.8	JF683499
NP	1565	45.4	A/equine/Richmond/1/2007	99.7	MT908913
NA	1406	41	A/equine/Meath/1/2007	99.7	JF683500
M	988	47.5	A/equine/Jammu and Kashmir/CMVL-LEH4/2008	99.8	JF683498
NS	890	40.6	A/equine/Hissar/CMVL-HSR4/2008	99.6	JF683501

HA gene alignment analysis of the A/equine/Otar/3/2007 strain showed three nucleotide substitutions relative to A/equine/Richmond/1/2007 strain. When the amino acid sequences of our isolates were aligned with the strain A/equine/Switzerland/P112/2007 six amino acid substitutions (positions 43D-V, 100G-R, 123G-E, 209M-T, 238L-P, 265I-V) were seen.

**FIG 1:**
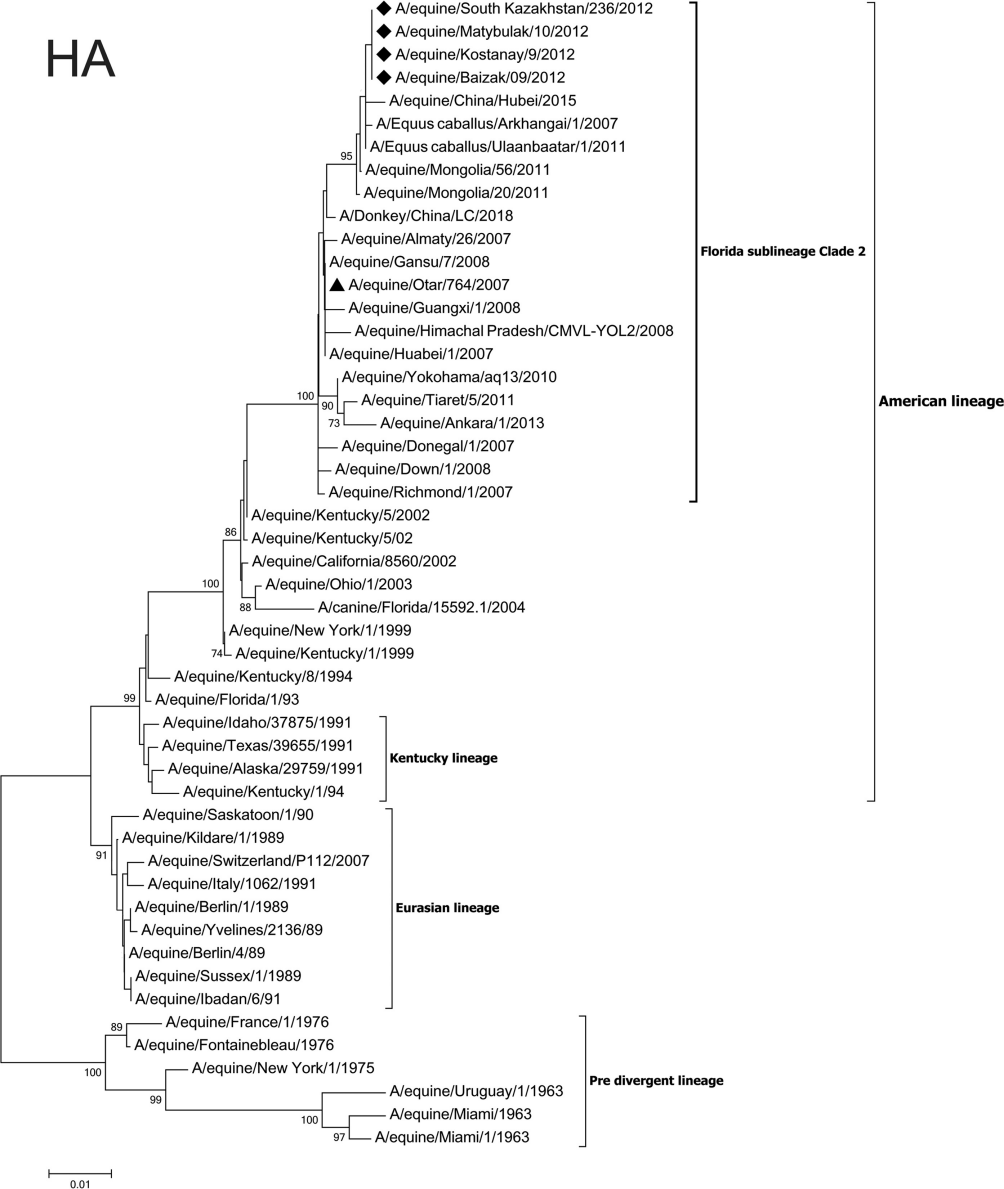
Phylogenetic tree for HA gene of the strain A/equine/Otar/3/2007 (H3N8). The evolutionary history of the HA gene was inferred using the neighbor-joining method. The percentage of replicate trees in which the associated taxa clustered together in the bootstrap test (10,000 replicates) are shown next to the branches (12). The tree is drawn to scale, with branch lengths in the same units as those of the evolutionary distances used to infer the phylogenetic tree. The evolutionary distances were computed using the Kimura 2-parameter method and are in the units of the number of base substitutions per site. Evolutionary analyses were conducted in MEGA7 (13, 14). The location of the sequence reported here is indicated with a black triangle. Rhombuses mark Kazakhstan strains of equine influenza virus isolated in 2012 (8).

Phylogenetic analysis of the HA protein of our A/equine/Otar/3/2007 strain clustered the virus among the American lineages and in particular the Florida sublineage clade II. Phylogenetic trees constructed with the NJ method using the HA sequence are shown in Fig. 1 (11).

### Data availability.

Complete genome sequence of strain A/Equine/Otar/3/2007 (H3N8) was published (deposited) in GenBank under the following numbers: MT908915, MT908914, MT965694, JF683499, MT908913, JF683500, JF683498, and JF683501.
